# Supplementary material for: Modeling glioblastoma heterogeneity as a dynamic network of cell states
Source: Mol Syst Biol. 2021 Sep 16;17(9):e10105. doi: 10.15252/msb.202010105 (PMC8444284; doi:10.15252/msb.202010105)
Supplement: Supplementary file 6 — Source Data for Figure 5 [file MSB-17-e10105-s004.zip › Figure5A_sourcedata/GSEA_3071/hallmarks_stateIII.GseaPreranked.1623742540963/HALLMARK_DNA_REPAIR.html]

Details for gene set HALLMARK\_DNA\_REPAIR[GSEA]

|  || Dataset | state33071 |
| Phenotype | NoPhenotypeAvailable |
| Upregulated in class | na\_pos |
| GeneSet | HALLMARK\_DNA\_REPAIR |
| Enrichment Score (ES) | 0.20216708 |
| Normalized Enrichment Score (NES) | 1.314122 |
| Nominal p-value | 0.14285715 |
| FDR q-value | 0.16155094 |
| FWER p-Value | 0.44 |
Table: GSEA Results Summary

  

Fig 1: Enrichment plot: HALLMARK\_DNA\_REPAIR      
 Profile of the Running ES Score & Positions of GeneSet Members on the Rank Ordered List

  

| PROBE | GENE SYMBOL | GENE\_TITLE | RANK IN GENE LIST | RANK METRIC SCORE | RUNNING ES | CORE ENRICHMENT || 1 | NME1 |  |  | 166 | 0.364 | -0.0020 | Yes |
| 2 | POLR2E |  |  | 192 | 0.344 | 0.0299 | Yes |
| 3 | POLR2J |  |  | 215 | 0.331 | 0.0611 | Yes |
| 4 | IMPDH2 |  |  | 253 | 0.309 | 0.0860 | Yes |
| 5 | POLR2I |  |  | 311 | 0.289 | 0.1036 | Yes |
| 6 | GTF2H5 |  |  | 331 | 0.284 | 0.1303 | Yes |
| 7 | GUK1 |  |  | 349 | 0.276 | 0.1567 | Yes |
| 8 | COX17 |  |  | 368 | 0.270 | 0.1821 | Yes |
| 9 | RBX1 |  |  | 418 | 0.254 | 0.1978 | Yes |
| 10 | BCAP31 |  |  | 542 | -0.256 | 0.1948 | Yes |
| 11 | REV3L |  |  | 693 | -0.264 | 0.1858 | Yes |
| 12 | RAD52 |  |  | 838 | -0.271 | 0.1791 | Yes |
| 13 | TAF1C |  |  | 903 | -0.274 | 0.1932 | Yes |
| 14 | RAD51 |  |  | 1025 | -0.281 | 0.1934 | Yes |
| 15 | PCNA |  |  | 1116 | -0.286 | 0.2022 | Yes |
| 16 | POLD1 |  |  | 1768 | -0.323 | 0.0713 | No |
| 17 | CLP1 |  |  | 1872 | -0.328 | 0.0814 | No |
| 18 | AGO4 |  |  | 1898 | -0.329 | 0.1117 | No |
| 19 | ADCY6 |  |  | 1982 | -0.334 | 0.1276 | No |
| 20 | SNAPC4 |  |  | 2016 | -0.336 | 0.1566 | No |
| 21 | TK2 |  |  | 2204 | -0.349 | 0.1476 | No |
| 22 | STX3 |  |  | 2395 | -0.365 | 0.1395 | No |
| 23 | BRF2 |  |  | 2439 | -0.368 | 0.1695 | No |
| 24 | DDB2 |  |  | 2834 | -0.402 | 0.1133 | No |
| 25 | POLH |  |  | 2869 | -0.405 | 0.1496 | No |
| 26 | PDE4B |  |  | 2878 | -0.406 | 0.1928 | No |
| 27 | ARL6IP1 |  |  | 3759 | -0.685 | 0.0436 | No |
Table: GSEA details [plain text format]

  

Fig 2: HALLMARK\_DNA\_REPAIR: Random ES distribution      
 Gene set null distribution of ES for **HALLMARK\_DNA\_REPAIR**
